# Case of a Giant Appendicolith

**DOI:** 10.7759/cureus.22034

**Published:** 2022-02-08

**Authors:** Cherisse A Rampersad, Fidel S Rampersad, Parasram R Ramraj, Vimal V Seetahal

**Affiliations:** 1 Radiology, Arima General Hospital, Arima, TTO; 2 Department of Radiology, The University of the West Indies, Port of Spain, TTO; 3 Department of Surgery, The Surgi-Med Clinic, San Fernando, TTO; 4 Department of Urology, St. Clair Medical Center, Port of Spain, TTO

**Keywords:** laparotomy in appendicitis, appendicolith, general surgery, appendicitis, giant appendicolith

## Abstract

Appendicoliths are calcified deposits located within the appendiceal lumen, usually measuring less than 1 cm in diameter. Appendicoliths greater than 2 cm in the largest diameter are uncommon and referred to as giant appendicoliths. Generally, patients with giant appendicoliths are asymptomatic, with these being detected incidentally on X-ray or computed tomography (CT). However, the presence of appendicoliths has been shown to be associated with an increased risk of developing appendicitis and is associated with more severe appendicitis. There is an increased incidence of appendicoliths in retrocecal appendices. This case report is of an adult male patient who presented with a three-day history of right iliac fossa pain, nausea, and decreased appetite. CT of the abdomen and pelvis showed acute appendicitis secondary to a calcified 3.1 cm giant appendicolith. Open appendicectomy was subsequently performed as the patient’s financial constraints hindered a laparoscopic approach. The clinical outcome was successful with no postoperative complications, and the patient was discharged the following day. The patient was reviewed six weeks post-laparotomy with no complaints and was discharged from the surgical outpatient clinic.

## Introduction

Acute appendicitis can be caused by gastrointestinal infections or by appendicoliths, with the latter accounting for 10% of cases [1]. When appendicoliths are present, perforation or abscess formation is a more common sequela of appendicitis. Very few cases of giant appendicoliths have been reported in the literature, with the largest measuring 3.5 cm in diameter [2].

## Case presentation

**Figure 1:**
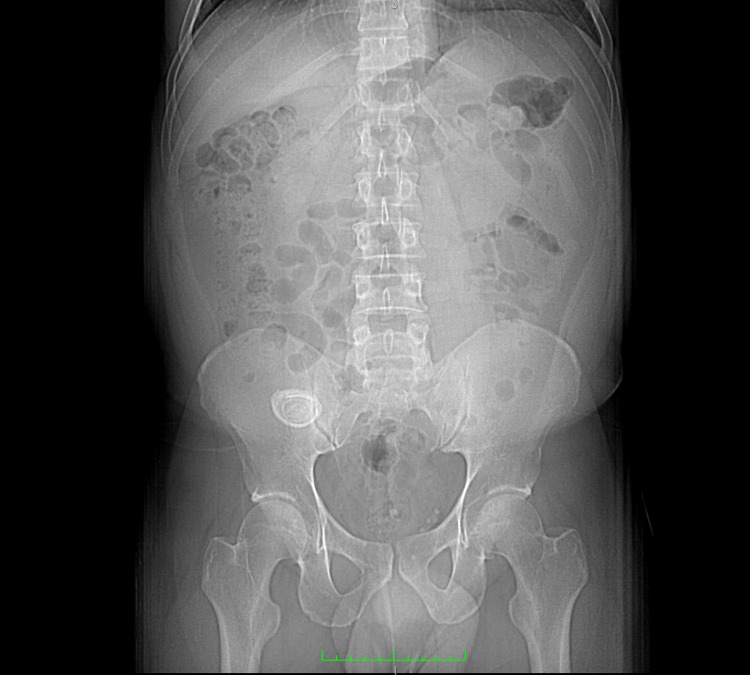
CT image showing an ovoid calcific density within the right iliac fossa, consistent with an appendicolith. CT: computed tomography

**Figure 2:**
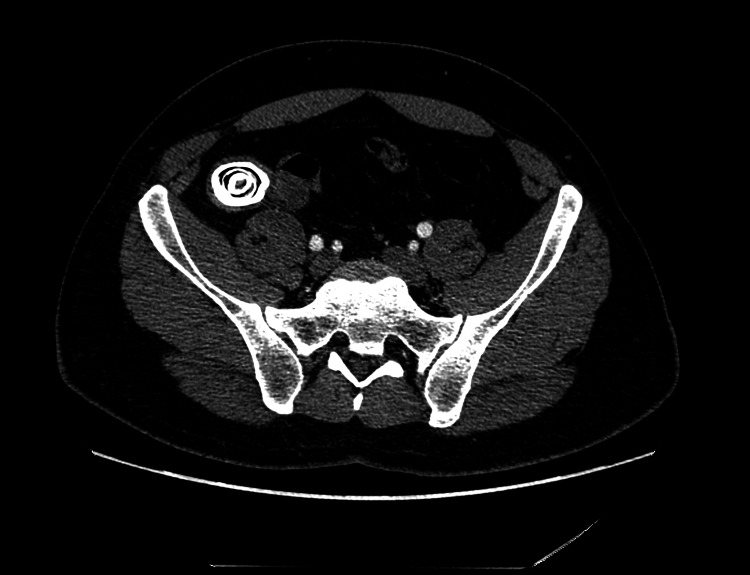
Axial postcontrast CT shows the lamellated appearance of the giant appendicolith within the right lower quadrant, with mild appendiceal mural thickening, indicative of mild acute appendicitis. CT: computed tomography

**Figure 3:**
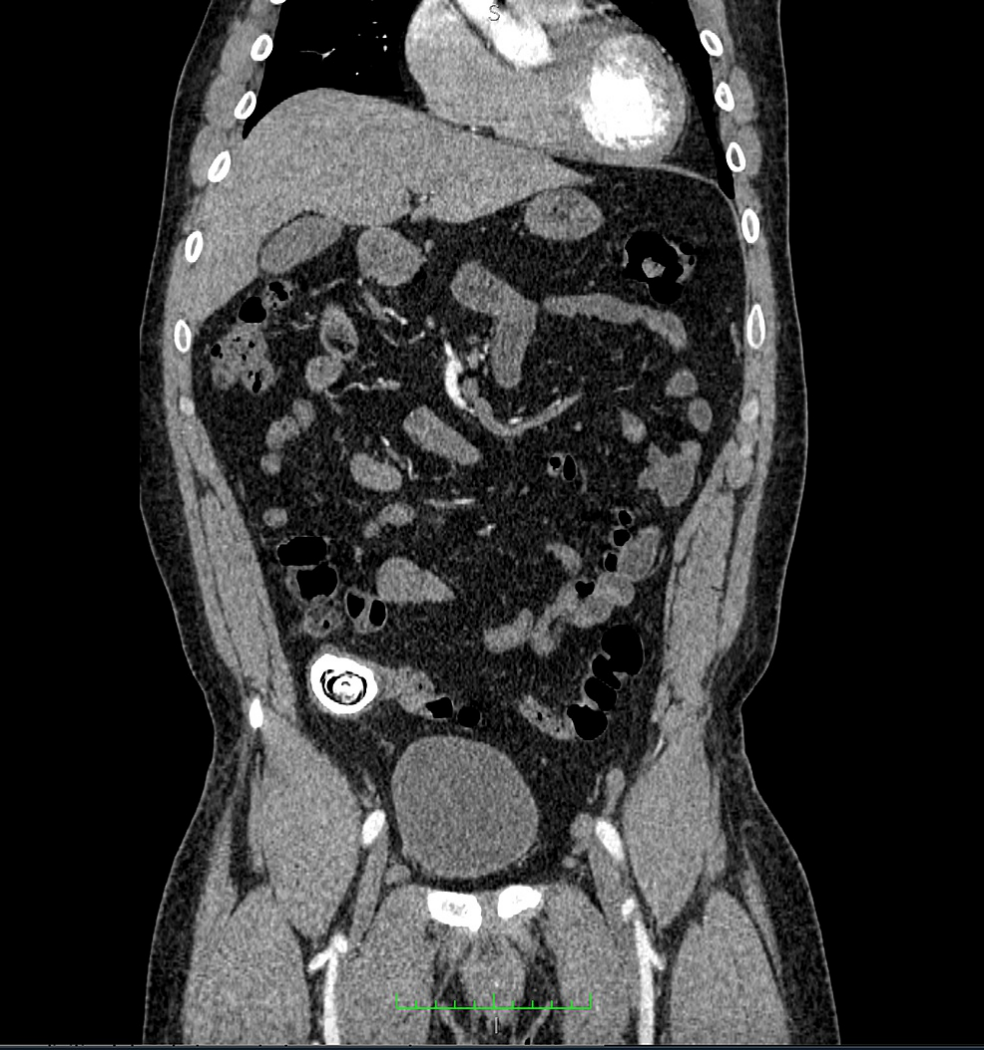
Coronal post IV contrast CT showing giant appendicolith within the right lower quadrant with periappendiceal fat stranding. No evidence of bowel obstruction or periappendiceal collection can be seen. CT: computed tomography

**Figure 4:**
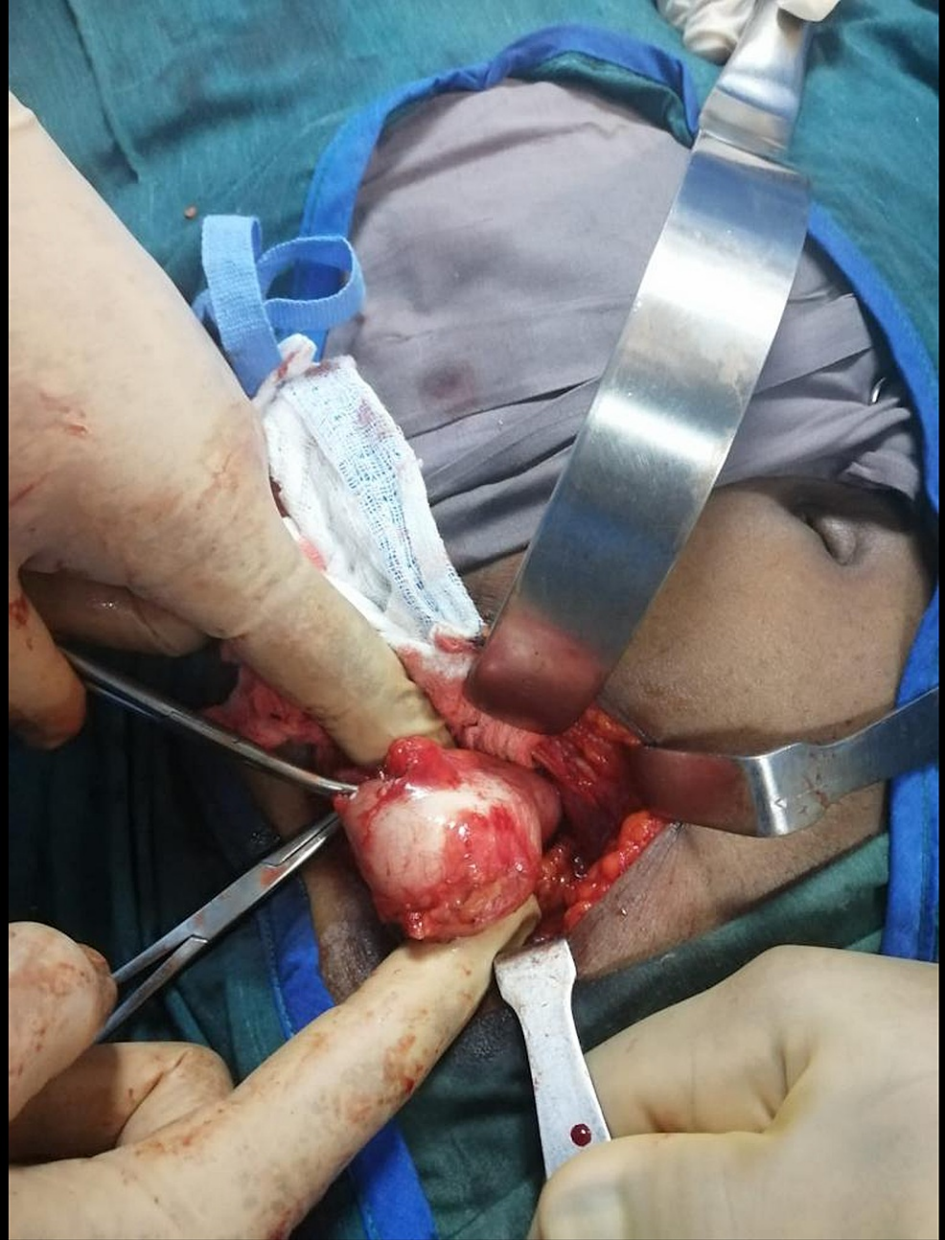
Intraoperative image showing the appendicolith being extracted.

A 48-year-old male patient with no known comorbidities and a history of conservative management of appendicitis in 2015 presented with a three-day history of right iliac fossa pain, with loss of appetite and nausea. On examination, Mc Burney’s sign was positive, and an ultrasound was performed which demonstrated a dilated appendix. Computed tomography (CT) of the abdomen and pelvis was performed, which showed a fluid-filled dilated appendix, periappendiceal fat stranding, and a 3.1 cm giant appendicolith, with no evidence of perforation or abscess formation (Figures 1-3). The patient subsequently underwent a successful open appendicectomy with an uneventful postoperative course (Clavien-Dindo score 1) (Figure 4).

## Discussion

Appendicoliths are solid, often calcified, deposits within the appendiceal lumen composed of firm feces and minerals. They are usually small (less than 1 cm) but can become larger than 2 cm, which are then termed giant appendicoliths. The largest reported giant appendicolith measured 3.5 cm in 2005 [1,3].

There is wide discordance regarding the prevalence of appendicoliths in appendicitis. While historical data depict a prevalence of 33-44%, contemporary evidence estimates a prevalence of approximately 1.5-15% [4]. Generally, appendicoliths are found more commonly in male patients and the retrocecal appendix [3]. Appendicitis secondary to an appendicolith occurs due to proximal luminal obstruction, with the subsequent rise in intraluminal pressure and vascular congestion causing luminal wall ischemia [5].

Giant appendicoliths are generally asymptomatic unless there is luminal obstruction. Patients present with typical features of appendicitis, such as colicky right lower quadrant abdominal pain progressing to guarding and rebound tenderness [5]. Appendicoliths may also be a cause of atypical signs and symptoms in appendicitis, such as appendicitis clinically masquerading as ureteral colic [6].

Appendicoliths are usually found incidentally on imaging studies and have a diagnostic sensitivity of 65% and specificity of 86% when detected on CT [5]. Diagnosis of appendicitis is mostly clinical with radiological imaging used to provide more diagnostic accuracy or to confirm the diagnosis when uncertain.

Giant appendicoliths can be managed conservatively when patients are asymptomatic or when surgery may impose greater risk to the patient [1]. Laparoscopic appendectomy is the most effective surgical treatment as well as treatment of choice as it is associated with a lower incidence of wound infection and post-intervention morbidity, shorter hospital stay, and better quality of life scores compared to open appendectomy, which was employed in this case where the appendicolith was complicated by acute appendicitis and emergent surgery was needed. Very few studies have described the use of endoscopic extraction as a treatment option with successful outcomes [1].

## Conclusions

Giant appendicoliths are rare and usually asymptomatic. However, when complicated, they are often associated with acute appendicitis with increased risk of perforation, for which urgent appendectomy is warranted.
